# Skin moisture measurement on stripping and pasted water by a handy‐type electrostatic sensor

**DOI:** 10.1111/srt.13125

**Published:** 2021-11-09

**Authors:** Akira Kimoto, Fumiaki Yonekawa, Tomoyuki Kawasoe

**Affiliations:** ^1^ Faculty of Science and Engineering Saga University 1 Honjo‐machi Saga 840‐8502 Japan; ^2^ Center for Education and Innovation Sojo University 4‐22‐1 Ikeda, Nishi‐ku Kumamoto City 860‐0082 Japan

**Keywords:** contact‐and‐release, electrostatic induction, handy‐type sensor, moisture

## Abstract

**Background:**

There is a need to develop a new device to evaluate and monitor the condition of human skin and make it possible to measure the skin in the diary because the current accuracy of water content measurement in the stratum corneum by capacitance and conductance measurement sensor that are used as the gold standard is insufficient.

**Materials and methods:**

The electrostatic sensor is composed of a thin silicone gum sheet and a copper film. In the experiment, skin conditions on six positions such as the forearm, upper arm, and face of a test subject before and after tape stripping are measured by the electrostatic sensor and a commercial sensor. Skin conditions on the forearm of five subjects before and after pasted distilled water were measured by their sensors.

**Results:**

The voltages measured by the electrostatic sensor and moisture measured by a commercial sensor are increased with *P* < 0.01 before and after skin stripping. There were increases in the voltage and the moisture with *P* < 0.01 before and after pasted distilled water.

**Conclusion:**

It is suggested that it is possible to measure the moisture on the upper layer of the skin by the electrostatic sensor.

## 1 INTRODUCTION

It is important and valiant to evaluate the water content of the skin, especially, the stratum corneum in the field of skin physiology and cosmetics. Several instrumentations have been developed and used for evaluating and monitoring the skin condition. Contact type instruments such as the Corneometer (Courage+Khazaka Electronic GmbH, Köln, Germany) and moisture checker (Scalar Corp, Tokyo, Japan) based on capacitance measurement and Skicon (IBS Corp., Hamamatsu, Japan) of conductance measurement are widely used as the gold standard for skin water content measurement.^1^, ^2^, ^3^, ^4^ Contactless instruments such as the attenuated total reflection‐Fourier transform infrared spectroscopy and the confocal Raman spectrometer are also used for evaluating the stratum corneum.^5^, ^6^, ^7^, ^8^ In addition, a number of contact and contactless new devices for skin water content measurement have been researched.^9^, ^10^, ^11^, ^12^, ^13^ There is now a need to develop a new device that evaluates the condition of human skin and makes it possible to monitor the skin in the diary life because the current accuracy of water content measurement in the stratum corneum by capacitance and conductance measurement sensors that are used as the gold standard is insufficient.^14^

The final purpose of our study is to develop a new sensing device for skin condition evaluation including the depth profiling water content. A new sensing method based on the electrostatic induction for this purpose was proposed^15^ although it has been used for material discrimination and human motion measurement.^16^, ^17^ In the experiments, it is demonstrated that the proposed sensor makes it possible to measure the water content in more surface layers than can be measured when using a commercial sensor.^18^ However, it was difficult to measure freely the skin condition such as upper arm and face since the sensor was moved using the industrial robot arm fixed on the floor. In this paper, a handy‐type electrostatic sensor that makes it easier to measure freely is evaluated. In the experiments, the skin conditions on six areas such as the upper arm, the forearm, and the face of a volunteer were measured before and after stripping using both the electrostatic and commercial sensors. In addition, the skin conditions on the forearm of five subjects were measured using both sensors before and after filter paper containing distilled water was pasted on the skin.

## 2 PRINCIPLE

**FIGURE 1:**
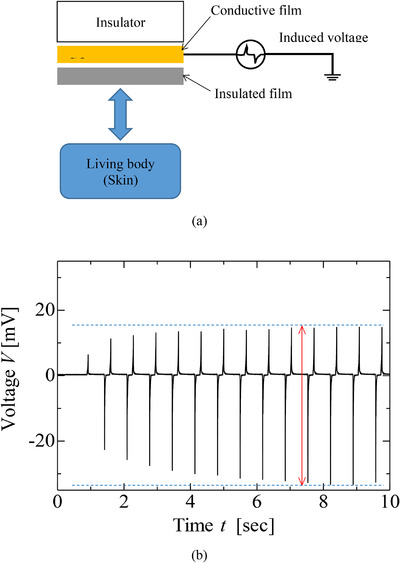
Sensing principle of electrostatic sensor. (A) Schematic diagram illustrating the measurement principle. (B) An example of voltage waveform measured by the sensor

Figure 1A shows a schematic diagram of the principle for skin water content sensing using the electrostatic sensor. The electrostatic sensor consists of insulating and conductive films arranged on an insulated base. The induced current flows through the conductive film as a result of the electrostatic effect, while the electrostatic sensor repeatedly contacts and then releases the skin.^15^ The current is dependent on the skin's water content. Therefore, the water content is measured using the electrostatic sensor. In this sensor, the current is converted to the voltage by I‐V converter. Figure 1B shows the voltage waveforms that were obtained using the electrostatic sensor. It was found that the amplitude of the peak voltage increases and saturates after approximately ten contact‐and‐release cycles (8 s) because of the electrostatic effect. The peak voltage of the voltage waveform was used for evaluating the electrostatic sensor since the peak voltage is changed by the skin's water content.

## 3 EXPERIMENTAL SECTION

### 3.1 Measurement system

**FIGURE 2:**
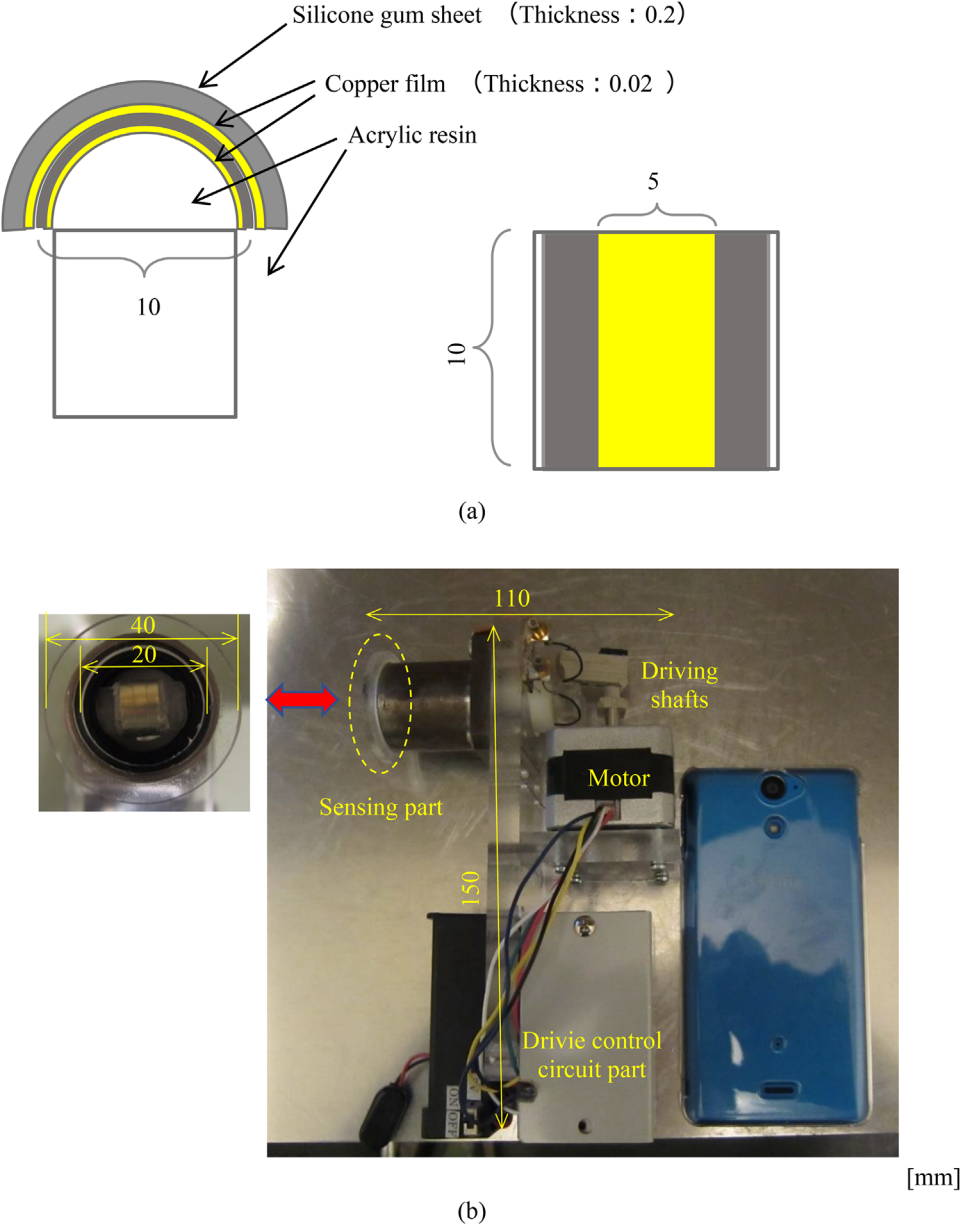
Schematic diagram of handy‐type electrostatic sensor. (A) Schematic diagram of sensing part which consists of copper film and silicone gum sheet. (B) Photograph of handy‐type electrostatic sensor comparing the smartphone

Figure 2 shows a schematic diagram of the handy‐type sensor that was used to perform skin water content measurements. Figure 2A shows a schematic diagram of the sensing part. A copper film (8 mm × 20 mm × 0.02 mm) that was connected to the ground was attached to an acrylic semi‐column with a diameter of 10 mm and length of 10 mm that acted as a base. The copper film (4 mm × 16 mm × 0.02 mm) that measures the induced current was pasted onto the base using an insulating film (polyethylene naphthalate (PEN) film, 8 mm × 20 mm × 0.05 mm). In addition, a silicone sheet (10 mm × 20 mm × 0.2 mm) was pasted onto the copper film. Figure 2B shows a photograph of the proposed sensor (with dimensions of 150 mm × 110 mm × 50 mm). The sensing part was attached to the front end of an insulated pipe (ϕ20 mm) inserted into the flanged linear bushing (LHFS20) as the sensing part was straightly moved to the target. The pipe was connected to the front end of a stepping motor (ST‐42BYG20) using the driving shafts and bearings for converting the rotation to the linear movement. In this way, the surface of the sensing part was moved from +1 mm to −9 mm, where the contact point between the top of the sensing part and the skin was defined as 0 mm. The motor's speed is controlled via the circuit. When the acrylic circular plate (ϕ40 mm) that was pasted to the front end of the flanged linear bushing was arranged on the subject's skin, the sensing part was repeatedly placed in contact with and released from the skin by operating the motor. The induced current was converted into a voltage and measured.

**FIGURE 3:**
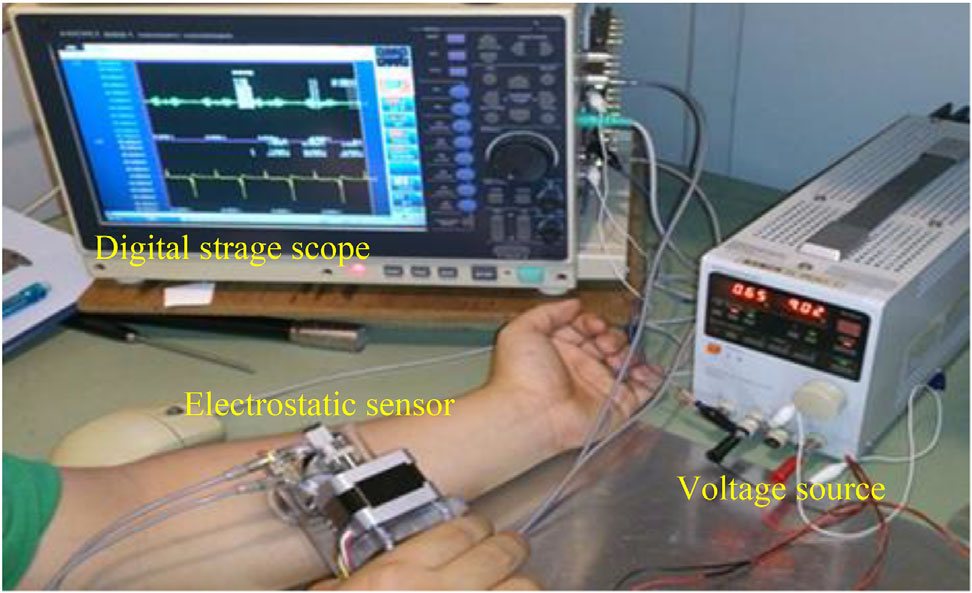
Photograph of measurement system in which skin water content at forearm was measured by the electrostatic sensor

Figure 3 shows the measurement system used in the experiments. The waveforms were measured using a digital storage oscilloscope with 12‐bit resolution and 500 Hz sampling frequency (HIOKI 8861 Memory Hicorder, HIOKI Corp., Nagano, Japan), and the measured data were then stored in a personal computer. The voltage waveforms were measured over 10 s of contact‐and‐release cycles, during which the amplitude of the waveform remained relatively stable.

### 3.2 Skin stripping experiment

**FIGURE 4:**
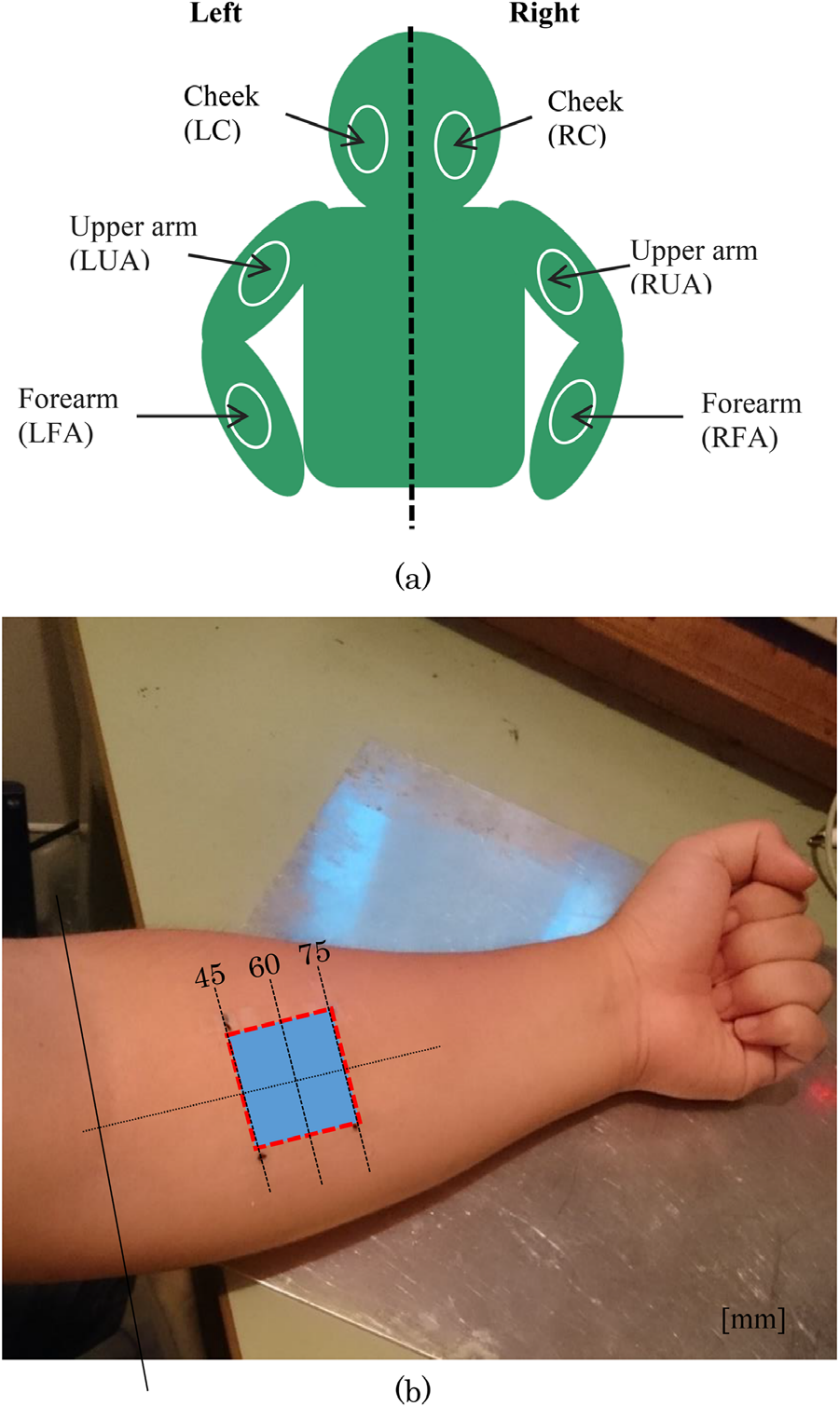
Experimental setup. (A) Six areas of skin stripping: cheek (LC, RC), upper arm (LUA, RUA), and forearm (LFA, RFA) on both sides. (B) Area on the forearm on which distilled water was pasted

Figure 4A shows a schematic diagram that illustrates the six stripping areas. The voltage was measured by the electrostatic sensor and the moisture was measured by the commercial sensor (Moisture checker MY‐808S, Scalar Corp., Tokyo, Japan) that is based on capacitance measurement and has a sensing area of approximately 1 cm^2^ ^4^, ^9^ before and after the stripping procedure. The experiment was carried out as follows. First, the healthy volunteer (a male subject in his forties) sat in a chair in a room under constant temperature (24°C) and humidity (20% ± 5%) conditions for 30 min after the sensing areas on the body had been washed with soap as the initial condition. Subsequently, the voltage and the moisture were measured by the electrostatic sensor and the commercial sensor, respectively, at each area to provide the initial values. Next, each area was stripped ten times using tape (Micropore™ Surgical Tape, 3 M Japan Ltd., Tokyo, Japan). After three minutes from that, the voltage was measured using the electrostatic sensor and the moisture was measured using the commercial sensor. The voltage and the moisture were both measured at least three times for evaluating the measurement accuracy. Informed consent based on the Declaration of Helsinki was obtained from the subject before the experiment.

### 3.3 Experiment using pasted distilled water

Figure 4B shows a schematic diagram of the experimental method in which distilled water was pasted on the subject's arm. The voltage and the moisture in an area on the forearm that is located 60 mm away from the subject's elbow were measured using the electrostatic sensor and the commercial moisture sensor, respectively. The experiment was carried out as follows. First, the volunteer sat on the chair in a room with the same constant temperature (24°C) and humidity (20% ± 5%) conditions for 30 min after their forearm had been washed with soap as the initial condition. The voltage and the moisture were subsequently measured using the electrostatic sensor and the commercial sensor, respectively, at the sensing area on the volunteer's forearm to acquire the initial values. Next, filter paper (30 mm × 30 mm, No. 131, Advantec, Toyo Roshi Kaisha Ltd., Tokyo, Japan) that was filled with 0.3 ml of distilled water was pasted on the sensing area on the subject's skin for 5 min to change their skin's water content. After another 5 min from the time at which the paper was removed, the voltage was measured using the electrostatic sensor and the moisture was measured using the commercial sensor three times each. As for this experiment, 13 times by five volunteers (a man in his 40s, three times, and two men (three times per man) and two women (twice per woman) in their 20s) were performed. Informed consent based on the Declaration of Helsinki was obtained from the subjects before the experiments.

## 4 RESULTS AND DISCUSSION

### 4.1 Experimental results of the skin ‐tripping procedure

**FIGURE 5:**
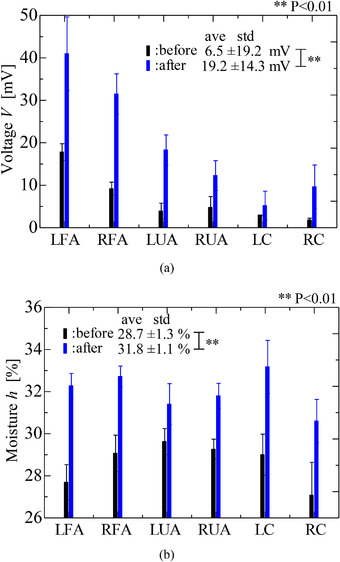
Experimental results of stripping (A) voltage measured by electrostatic sensor and (B) moisture measured by commercial sensor before and after stripping. Voltages and moisture measured on six areas are evaluated by a paired *t*‐test

Figure 5 shows the results obtained for the peak voltages using the electrostatic sensor and for the moisture using the commercial sensor, both before and after skin stripping. The horizontal axis shows the six sensing areas, including the forearm (LFA), the upper arm (LUA), and the cheek (LC) on the left side and the corresponding areas on the right side (RFA, RUA, and RC, respectively). The voltage and moisture results are shown as average values with standard deviations after they were measured at least three times.

From Figure 5, it was found that the average values of the voltages and the moisture measured at the sensing areas respectively increased with approximately 12 mV (3 times) and 3% after skin stripping. In addition, it was found that the measured voltages and moisture on six areas were respectively differed via a paired *t*‐test at a significance probability of 0.01. Therefore, the change in the skin condition caused by the stripping was detected using the electrostatic sensor. It was found that the surface layer of the skin was stripped and the inside of stratum corneum that was more hydrated than the surface layer was barely formed. It was estimated that the increase in the moisture detected by the commercial sensor was caused by the inside of the stratum corneum which was generally more hydrated than the surface.^19^ It was also supposed that the upper layer after stripping was exposed to dry condition (20% ± 5%) and became the drier condition.^20^ The results supported the supposition that the increase in the voltage was caused by the surface of the skin being in a drier condition than it had been before the stripping procedure because the voltage was reduced in the case where the skin condition showed increased moisture.^18^ Therefore, it was believed that the electrostatic sensor had measured a different surface area of the skin to that measured using the impedance moisture sensor, although the reason for this was not clearly addressed.

### 4.2 Experimental results after pasting of distilled water on filter paper

**FIGURE 6:**
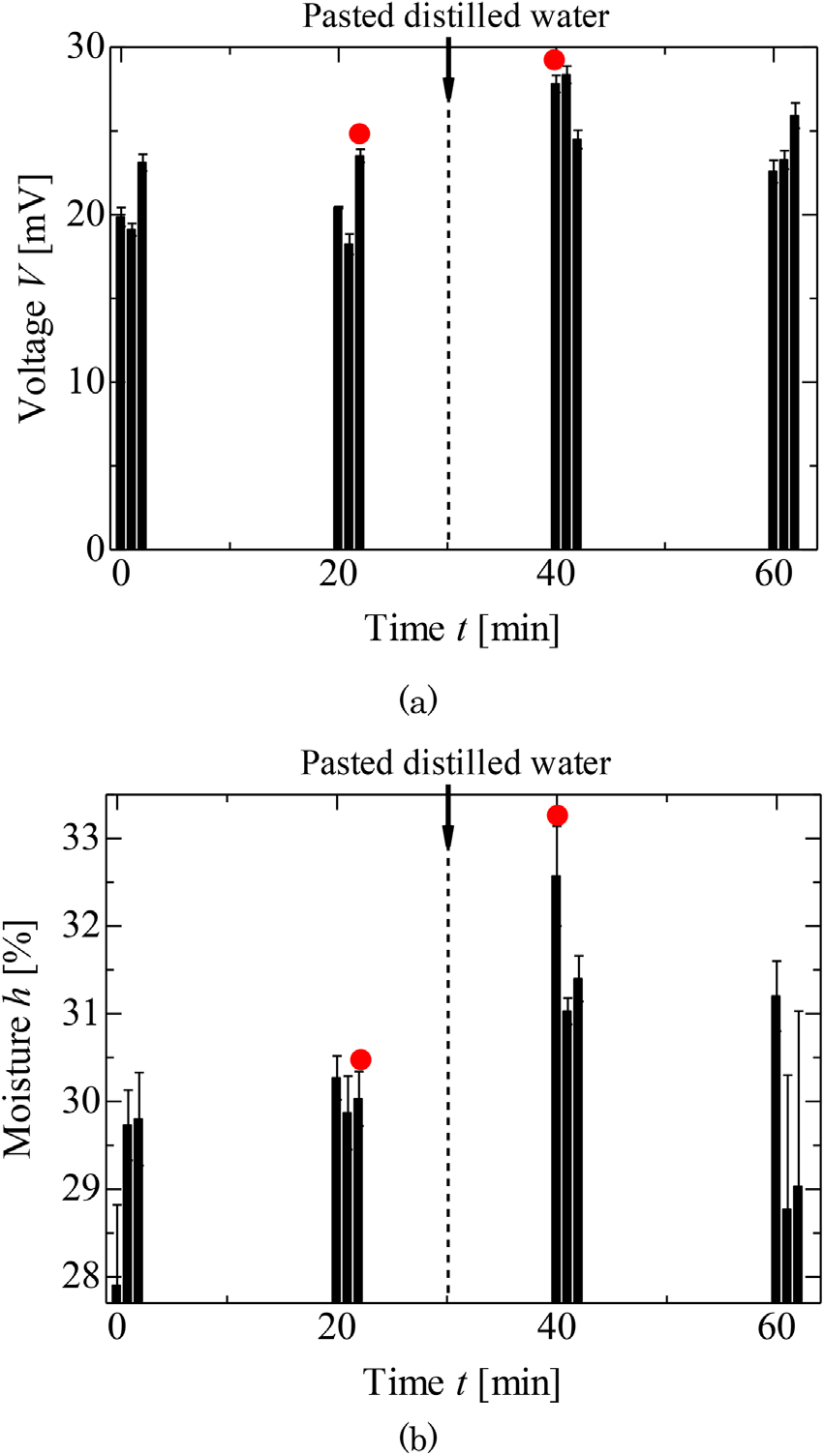
Experimental results of pasted distilled water on the forearm. (A) Voltage measured by electrostatic sensor. (B) Moisture measured by commercial sensor

**FIGURE 7:**
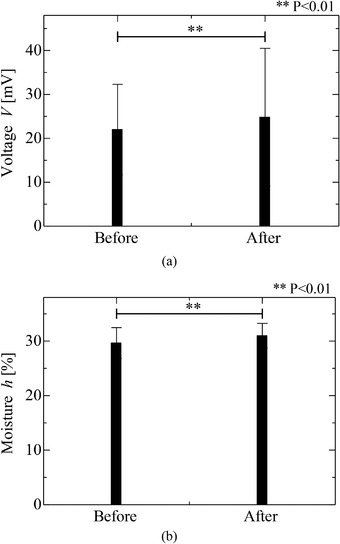
Comparison of the proposed sensor with the moisture sensor before and after pasting of the distilled water via a paired *t*‐test based on thirteen results of five volunteers. (A) Voltages measured by electrostatic sensor. (B) Moisture measured by commercial sensor

Figure 6 shows the results obtained from one volunteer for the peak voltages measured via the electrostatic sensor and the moisture measured via the commercial sensor before and after the filter paper containing the distilled water was pasted on their skin for 30 min. The results indicated that the voltages and the moisture were increased by the pasting of the distilled water. Therefore, the change induced in the skin condition by the distilled water was detected using the electrostatic sensor, as well as in the stripping experiment. Figure 7 shows the thirteen results acquired for the voltages and moisture contents of the five volunteers, where the results acquired before and after the distilled water was pasted (the red color dotted circles in Figure 6) are compared. From these results, it was found that the voltages measured using the electrostatic sensor and the moisture measured using the commercial sensor differed via a paired t‐test at a significance probability of 0.01. Therefore, it was found that the change of water content was detected by using the handy‐type electrostatic sensor. It was estimated that the increase in moisture measured by commercial sensor was caused by which the pasted water penetrated to the skin with the depth of 10 μm.^21^ The voltage also increased as well as that measured using the commercial moisture sensor, although it was estimated that the voltage was reduced by involving the skin with the water so that the skin moisture increased.^18^ The increase in the voltage was caused by the upper layer of the skin being in drier conditions as well as that of the skin stripping.^20^ Therefore, it was found that the electrostatic sensor has the potential for sensing the moisture on the upper layer of the skin, although phenomena such as the effect of the electrostatic sensor in the direction of the depth of the skin must be addressed clearly.

In these experiments, the measurement accuracy was insufficient. The inaccuracy was mainly caused by the unstable contact face and the angle between the sensor and the skin. Therefore, future work will involve stabilizing the contact force and contact angle by improving the mechanism of linear movement.

## 5 CONCLUSIONS

In this paper, a handy‐type electrostatic sensor was evaluated. In the experiments, the voltage measured using the electrostatic sensor and the moisture measured using a commercial sensor at six different sensing areas on the skin, including the upper arm, forearm, and cheek of the subject, were measured before and after skin stripping. In addition, the skin on the forearm was also measured using the proposed and commercial sensors before and after filter paper containing distilled water was pasted to the skin. The results indicated the electrostatic sensor has the potential for detecting the moisture on the upper layer of the skin.
